# Radiolaria Divided into Polycystina and Spasmaria in Combined 18S and 28S rDNA Phylogeny

**DOI:** 10.1371/journal.pone.0023526

**Published:** 2011-08-10

**Authors:** Anders K. Krabberød, Jon Bråte, Jane K. Dolven, Randi F. Ose, Dag Klaveness, Tom Kristensen, Kjell R. Bjørklund, Kamran Shalchian-Tabrizi

**Affiliations:** 1 Microbial Evolution Research Group (MERG), Department of Biology, University of Oslo, Oslo, Norway; 2 Natural History Museum, University of Oslo, Oslo, Norway; 3 Department of Molecular Biosciences, University of Oslo, Oslo, Norway; 4 Department of Geosciences, University of Oslo, Oslo, Norway; Université Paris Sud, France

## Abstract

Radiolarians are marine planktonic protists that belong to the eukaryote supergroup Rhizaria together with Foraminifera and Cercozoa. Radiolaria has traditionally been divided into four main groups based on morphological characters; i.e. Polycystina, Acantharia, Nassellaria and Phaeodaria. But recent 18S rDNA phylogenies have shown that Phaeodaria belongs within Cerocozoa, and that the previously heliozoan group Taxopodida should be included in Radiolaria. 18S rDNA phylogenies have not yet resolved the sister relationship between the main Radiolaria groups, but nevertheless suggests that Spumellaria, and thereby also Polycystina, are polyphyletic. Very few sequences other than 18S rDNA have so far been generated from radiolarian cells, mostly due to the fact that Radiolaria has been impossible to cultivate and single cell PCR has been hampered by low success rate. Here we have therefore investigated the mutual evolutionary relationship of the main radiolarian groups by using the novel approach of combining single cell whole genome amplification with targeted PCR amplification of the 18S and 28S rDNA genes. Combined 18S and 28S phylogeny of sequences obtained from single cells shows that Radiolaria is divided into two main lineages: Polycystina (Spumellaria+Nassellaria) and Spasmaria (Acantharia+Taxopodida). Further we show with high support that Foraminifera groups within Radiolaria supporting the Retaria hypothesis.

## Introduction

Radiolarians are holoplanktonic protists with a worldwide distribution throughout the oceans [1], [2]. The traditional taxonomic scheme of radiolarians has been based on the shape and morphology of a central capsule and on the morphology of their skeletons [3]. With the central capsule in common, radiolarians have been divided into four groups; Nassellaria and Spumellaria (together they define the group Polycystina) are united by their siliceous skeleton, Acantharia is unique in having a skeleton made of strontium sulfate, and Phaeodaria which have a skeleton of organic substances intermixed with silica [1], [2], [3].

When the first molecular phylogenies of radiolarians were produced it became clear that Radiolaria was not a natural monophyletic group [4], [5]. Phaeodaria was moved from Radiolaria to its sister group Cercozoa, and the polycystines were found to be paraphyletic with Nassellaria and the colonial and naked spumellarians forming a monophyletic group together with Acantharia [4], [6]. The picture was further complicated when the heliozoan species *Sticholonche zanclea* (Taxopodida) was shown to belong to Radiolaria as sister to Spumellaria [5], [7]. Cavalier-Smith [8] had already grouped Acantharia and Taxopodida together in the sub-phylum Spasmaria based on their shared presence of spasmin-like myonemes. The addition of environmental sequences of 18S has not changed the overall phylogenetic relationships, although new undescribed groups have been discovered [9], [10], [11]. However, the relationship between the main groups of radiolarians is still uncertain, mainly because of weak statistical support in molecular phylogenies of the 18S gene.

Radiolaria belongs to the supergroup Rhizaria together with Cercozoa, Foraminifera and a few other groups including Haplosporidia, Gromia and Phytomyxea [12], [13]. Molecular phylogenies of Rhizaria have been notoriously difficult to resolve and the monophyly of the supergroup was only recently confirmed by molecular phylogenies [14]. But the relationship between the different groups is still uncertain. Especially Foraminifera has been difficult to place, and whether this is a sister group to Gromia or Radiolaria is still a subject of debate [12], [15], [16].

In several of the 18S rDNA phylogenies Foraminifera clustered within the radiolarians [6], [12], [17]. However as Foraminifera are known to have extremely aberrant ribosomal genes this was regarded as an artifact resulting from long branch attraction [12]. It has therefore been uncertain whether Foraminifera are more closely related to Radiolaria or to Cercozoa. A recent phylogenomic analyses of Rhizaria [18] lends support to the Retaria hypothesis: that Foraminifera is sister to Radiolaria [19]. However, as Acantharia was the only representative of Radiolaria in that study, it is unclear whether Foraminifera should be included in the Radiolaria or whether it is one of the closest sister groups [18].

The main reason the Radiolarian phylogeny and its relationship to Foraminifera is still not resolved is the lack of gene sequences from Radiolaria that are applicable for phylogenetic inferences. Radiolarians are currently impossible to grow in culture as no one has so far succeeded in bringing any radiolarian species through a reproduction cycle. Therefore, mainly single-cell PCR has been used for amplification of 18S rDNA on identified Radiolaria. Despite the number of attempts at performing single-cell PCR the relative number of publicly available 18S rDNA sequences remains low [6], [7], [20], [21], [22], [23].

The aim of this study is to resolve the relationship between the major groups of Radiolaria, e.g. Acantharia, Taxopodida, Spumellaria and Nassellaria, by phylogenies based on concatenated 18S and 28S rDNA gene alignments. To obtain both genes from a single individual of Radiolaria we combined single cell whole genome amplification (SCWGA) with gene-targeted PCR on species of all major radiolarian groups as well as Phaeodaria. The revised phylogeny is used for interpretation of the Radiolaria and Foraminifera relationship and the evolution of cell structures among the Radiolaria lineages.

## Materials and Methods

### Sampling

**Figure 1:**
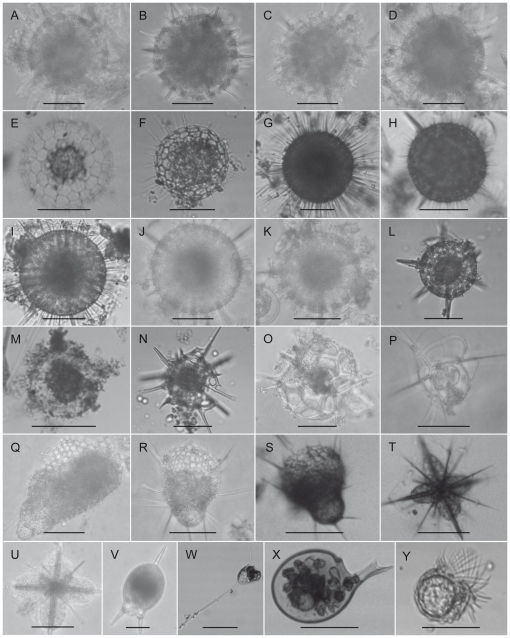
Light micrographs of the specimens studied. Pictures are taken directly from seawater samples in petri dishes under a Zeiss RA Compound microscope prior to washing. All scale bars are 50 µm. A) *Actinomma boreale* #43, B) *A. boreale* #47, C) *A. boreale* #72, D) *A. boreale* #79, E) *Cladococcus viminalis* #83, F) *C. viminalis* #302, G) *Hexaconthium gigantheum* # 9, H) *H. gigantheum* #12, I) *H. gigantheum* #293, J) *H. pachydermum* #71, K) *H. pachydermum* #86, L) *H. pachydermum* #294, M) *Phorticium pylonium* #245, N) *Streblacantha circumtexta #299,* O) *Ceratospyris hyperborea #134, P) Cladoscenium tricolpium* #49, Q) *Eucyrtidium calvertense* #129, R) *Lithomelissa setosa* #2, S) *L. setosa* #15, T) *Acanthonia nordgaardi* #16, U) *A. nordgaardi* #32, V) *Challengeron diodon* #18, W) *Medusetta archifera* #24, X) *Protocystis tridens #*143, Y) *Sticholonche zancela* #313.

Water samples were collected with a Juday-net in the innermost part of Sogndalsfjorden at the west coast of Norway (61° 12′ 30″ N, 07° 06 24″ E), in August 2009, March 2009 and January 2010 (see Table 1). Individual cells were photographed directly in subsamples of the nethaul (see Figure 1) and extracted using capillary isolation as described in [24]. After isolation each cell was individually washed and visible foreign material and extracellular debris was removed using microneedles. Following the physical washing each cell was rinsed separately in two droplets of sterile filtered water with the osmolarity adjusted with NaCl_2_ to 35 psu. Finally each cell was rinsed in one droplet of Milli-Q water (Millipore, Billerica, MA, USA) before storing at −80°C. To reduce the risk of airborne contamination, cleaning of cells was done inside a clean tent of transparent plastic film surrounding the microscope and dissecting area. For a more detailed description of sample site and sampling method see [25].

**Table 1:** Individual radiolarians sequenced in this study.

Type	Name	Individual #	rDNA gene		Accession #	Length (bp)	Sampling date	PCR primers	
			18S	28S				1. round	2. round
**Spumellaria**	*Actinomma boreale*	43	+	+	HQ651781	5122	09.03.2009	NSF4-3180R	NSF83-3180R
	*Actinomma boreale*	47	+	+	HQ651780	5044	09.03.2009	NSF4-3180R	NSF83-3180R
	*Actinomma boreale*	72	+	-	HQ651788	1680	09.03.2009	NSF4-1528R	NSF83-1528R
	*Actinomma boreale*	79	+	-	HQ651789	1626	09.03.2009	NSF83-1528R	
	*Cladococcus viminalis*	83	+	-	HQ651792	1516	09.03.2009	NSF4-1528R	NSF83-1528R
	*Cladococcus viminalis*	302	+	+	HQ651782	5214	25.01.2010	NSF4-3180R	NSF83-3180R
	*Hexacontium gigantheum*	9	+	-	HQ651795	1627	09.03.2009	NSF83-1528R	191F-1528R
	*Hexacontium gigantheum*	12	+	-	HQ651794	1630	09.03.2009	NSF83-1528R	191F-1528R
	*Hexacontium gigantheum*	293	+	-	HQ651796	1630	25.01.2009	NSF83-1528R	
	*Hexacontium pachydermum*	71	+	-	HQ651798	1629	09.03.2009	NSF83-1528R	191F-1528R
	*Hexacontium pachydermum*	86	+	+	HQ651784	4915	09.03.2009	NSF4-3180R	NSF83-3180R
	*Hexacontium pachydermum*	294	+	-	HQ651797	1630	25.01.2010	NSF83-1528R	
	*Phorticium pylonium*	245	+	+	HQ651783	3714	11.08.2009	NSF4-3180R	NSF83-3180R
	*Streblacantha circumtexta*	299	+	-	HQ651803	1692	25.01.2010	NSF4-1528R	NSF83-1528R
**Nassellaria**	*Ceratospyris hyperborea*	134	+	-	HQ651791	1715	09.03.2009	NSF4-1528R	NSF83-1528R
	*Cladoscenium tricolpium*	49	+	-	HQ651793	1672	09.03.2009	NSF4-1528R	NSF83-1528R
	*Eucyrtidium calvertense*	129	+	+	HQ651779	5070	09.03.2009	NSF4-3180R	NSF83-3180R
	*Lithomelissa setosa*	2	+	-	HQ651802	1793	09.03.2009	1F-1528R	
	*Lithomelissa setosa*	15	+	-	HQ651801	1792	09.03.2009	1F-1528R	
**Acantharia**	*Acanthonia nordgaardi*	16	+	-	HQ651787	1780	09.03.2009	NSF83-1528R	
	*Acanthonia nordgaardi*	32	+	+	HQ651786	4369	09.03.2009	NSF4-3180R	NSF83-3180R
**Taxopodida**	*Sticholonche zanclea* *	313	+	+	HQ651785	3845	25.01.2010	1F-3180R	Sticho1-3180R
**Phaeodaria**	*Challengeron diodon*	18	+	-	HQ651790	1597	09.03.2009	NSF4-1528R	NSF83-1528R
	*Medusetta archifera*	24	+	-	HQ651799	1041	09.03.2009	NSF4-1528R	NSF83-1528R
	*Protocystis tridens*	143	+	-	HQ651800	902	09.03.2009	NSF4-1528R	NSF83-1528R

*For *Sticholonche zanclea* a third round of PCR was needed to amplify the 18S and 28S rDNA genes. The primers used for this were Sticho1-28S_Rad3_R.

The species names are given in accordance to their morphology (see Figure 1) and clade affiliation given in accordance to classical taxonomy.

### Whole genome amplification and PCR

Cells were lysed in 3 µl of an alkaline buffer (0.4 M KOH, 01 mM EDTA, 0.1 M DTT) and heated to 95°C for 15 min to ensure that the central capsule would break. The alkaline lysis was neutralized with 3 µl of 0.4 M HCl. The entire reaction volume was subsequently used as template for whole genome amplification (WGA) by the Repli-g mini kit (Qiagen, Germantown, MD, USA) following the manufacturers instructions. Exceptions to the protocol were the use of in-house lysis and neutralization buffers instead of the kit-provided. The WGA was performed according to an optimized protocol to reduce the level of non-template amplified DNA; i.e. the amplification process was run for 4 hours at 30°C, then followed by 15 min at 65°C to deactivate the enzyme.

Of the approximately 60 µl WGA product, about 1 µl was used as template for PCR amplification of 18S rDNA genes. Trehalose was used as a PCR enhancer with a final concentration of 0.6 M [26]. The PCR was run in 25 µl reactions containing 1 µl of WGA template, 2.5 µl of 10X buffer, 200 µM dNTPs, 0.6 M trehalose, 2.875 µl Mili-Q water, 0.6 units DreamTaq DNA polymerase (Fermentas, Burlington, Canada) and 0.2 mM of forward and reverse primers (for the different primer combinations see Table 1 and 2). The PCR was run under the following conditions: 95°C for 2 min, followed by 35 cycles of 95°C for 30 s, annealing at 52–57°C for 30 s depending on the primers used, 72°C for 2 min for 18S rDNA and 6 min for 18S+28S rDNA. The final cycle was extended with an additional 10 min at 72°C to complete any unfinished fragments. 18S and 28S rDNA were amplified as one continuous fragment with forward primers matching the start of the 18S gene and reverse primers matching the end of the 28S gene. In instances where the PCR worked sub-optimally a semi-nested PCR strategy was employed, in which case 1 µl of the first PCR reaction mixture was used as a template with an identical amplification program as the first round, but with a new set of primers (see Table 1). Occasionally we amplified genes from symbionts or other associated organisms as well as the host gene, in which case the PCR products were cleaned using the Wizard SV gel and PCR Clean-Up System (Promega, Madison, WI, USA) and cloned using either the TOPO-TA kit (Invitrogen, Carlsbad, USA) or the pGEM-T Easy Vector System (Promega, Madison, WI, US) following the manufacturers instructions. PCR products and positive inserts were sequenced on a ABI 3730 DNA Analyzer using the ABI BigDye terminator v3.1 kit (Applied Biosystems, Foster City, CA, US).

**Table 2:** PCR primers used in this study.

Name	Direction	Gene	Sequence (5′–3′)	Length	Reference
3180R	Reverse	LSU	GGGTAAAACTAACCTGTCTCACGACGGTC	29	Ema Chao pers. comm.
28S_Rad3_R	Reverse	LSU	CGGTCTTCAAAGTTTTCATTTG	22	Designed for this study
Sticho1	Forward	LSU	TACATGCACGAAGGTCCAAC	20	Designed for this study
NSF4	Forward	SSU	CTGGTTGATYCTGCCAGT	18	http://bioinformatics.psb.ugent.be/webtools/rRNA/primers/NS_lst.html
NSF83	Forward	SSU	GAAACTGCGAATGGCTCATT	20	http://bioinformatics.psb.ugent.be/webtools/rRNA/primers/NS_lst.html
1528R	Reverse	SSU	TCCTTCTGCAGGTTCACCTAC	21	Medlin et al. (1988) [48]
1F	Forward	SSU	CTGGTTGATCCTGCCAG	17	Medlin et al. (1988) [48]
Spu_191F	Forward	SSU	GCGACTYACGAAGCCCTGTA	20	Yuasa et al. (2004) [49]

### Alignment construction

All sequences generated were checked for the presence of chimeras by using the program Key DNA Tools (http://keydnatools.com/). Two data sets were created, the first with 158 near full-length 18S rDNA sequences based on data sets used earlier [9], [11], [20]. The second dataset consisted of 27 taxa from which both 18S and 28S rDNA where available in GenBank, including the sequences produced in this study. The dataset contained representatives of the major Radiolaria clades and also representatives of Cercozoa, Foraminifera and Alveolata (outgroup). 18S and 28S of sufficient length were only available for three species of Foraminifera. The sequences where aligned using Opal [27] and manually checked with Mesquite v2.73 [28]. Ambiguously aligned characters were identified using Gblocks [29], with allowance for small final blocks (minimum length of block was set to 5), gaps were allowed in 50% of the sequences, and flanking positions were defined as half of the sequences +1. The final decision for inclusion or exclusion in the analysis was done manually using Mesquite v2.73 [28]. The final datasets, after exclusion of ambiguously aligned characters, contained 158 taxa and 1438 sites for the 18S rDNA alignment and 27 taxa and 4163 sites for the concatenated 18S and 28S rDNA alignment.

### Phylogenetic analyses

ML analyses were performed using the program RAxML v7.2.6 [30] with the general time reversible (GTR) model with a gamma distributed rate of variation across sites (Γ) and a proportion of invariable site (I), as selected by ModelTest [31]. The topology with the highest likelihood score out of 500 heuristic searches from randomly selected starting trees was chosen. Bootstrap scores were calculated from 500 pseudo-replicates using the best topology as starting tree.

The Bayesian inferences were performed with MrBayes v3.2.1 [32] using the GTR+Γ+I model. 10 independent analyses were done, each for 5.000.000 generations and with 3 Markov chain Monte Carlo chains in each run, two of which were heated, with a temperature set at 0.1. The runs were checked for convergence after the analysis was finished. The posterior probabilities were calculated after a burn-in of 25% of the initially sampled trees. Sequences generated in this study are deposited in GenBank (http://www.ncbi.nlm.nih.gov/genbank) with the accession numbers HQ651779-HQ651803. Fast evolving sites were calculated using the AIR package on the 18S dataset and the concatenated 18S+28S dataset [33]. 10% to 90% of fastest evolving sites (percentage of total rate variation) were removed with 10% intervals (Tables S1, S3 and S4). For the 18S+28S data this was performed both with and without Foraminifera in the alignment. Bayesian and ML analyses on the reduced alignments were performed as before. Phylogenetic analyses were also done on the 18S alignment after having removed colonial and naked spumellarians (Table S2) and Foraminifera to see the influence of these long branching groups on the general topology.

All phylogenetic analyses were run at the Bioportal (http://www.bioportal.uio.no) [33] or the Titan computer cluster at the University of Oslo.

## Results

### 18S rDNA phylogeny

**Figure 2:**
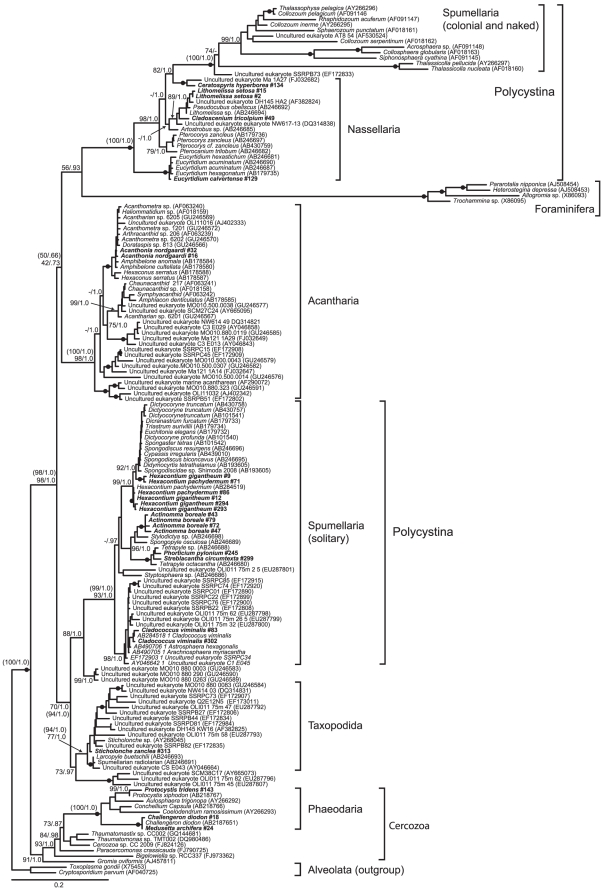
Maximum likelihood (ML) phylogeny of Radiolaria inferred from an 18S rDNA alignment consisting of 158 taxa and 1438 characters. Values at nodes represent bootstrap support values from ML analysis and Bayesian posterior probabilities (pp) (ML/pp). Filled circle indicate maximum support in both analyses. Only values above 70% and 0.85 pp are shown (except at a few backbone nodes) and only at selected nodes due to space constraints. Hyphen indicates values below the threshold. Values in parentheses indicate support values when the analyses were run without Foraminifera (ML/pp) and are only shown for the main groups.

18S rDNA sequences were obtained from 25 cells: 14 Spumellaria, 5 Nassellearia, 1 Taxopodida, 2 Acantharia and 3 Phaeodaria (for pictures and names of the species see Table 1 and Figure 1). The phylogenetic analysis of the 18S rDNA gene was highly congruent with several recent analyses (Figure 2) [9], [11], [20]. The phylogeny also confirms that the sequences obtained in this study actually arise from the host cell itself and not from potential prey or symbiotic organisms within the cell. The reconstructed tree strongly grouped together colonial and naked spumellarians (100%/1.0 pp). These were highly supported as a clade within Nasselaria (100%/1.0 pp). Removing fast evolving sites did not reduce the support for this clade (Table S1). These further clustered weakly as sister to Acantharia (50%/0.66 pp). When 80% of the fastest evolving sites were removed, the sisterhood of these two groups dissolved and Acantharia grouped weakly with Spumellaria and Taxopodida (42%, Table S1). Solitary and shell bearing spumellarians (Spumellaria) clustered together as a monophyletic group with high support (99%/1.0 pp). Taxopodida grouped with environmental sequences and the spumellarian *Larcopyle buetschlii* into a highly supported monophyletic clade and was sister to the solitary spumellarians (94%/1.0 pp). This position of *L. buetschlii* [also seen in 7,12] is to us a mystery as the morphology of *L. buetschlii* is very different from *Sticholoche* sp. When fast evolving sites were removed Taxopodida and Spumellaria remained grouped together, although the support for Taxopodida as a monophyletic group was weakened (see Table S1). Phaeodaria was well nested within the cercozoans and grouped together with maximum support (100%/1.0 pp). When Foraminifera was included they clustered strongly with the radiolarians (98%/1.0 pp) as sister to the assembly of Nassellaria and the colonial and naked spumellarians (56%/0.93 pp). After removing fast evolving sites the support values for the placement of Foraminifera peaked at 77% (Table S4). In addition a number of clades composed of only environmental sequences were recovered, these were largely the same as identified by Not et al. 2007 [9]. Removing naked and colonial spumellarians from the analysis did not change any of the support values for the major groupings notably (see Table S2).

### 18S + 28S rDNA phylogeny

**Figure 3:**
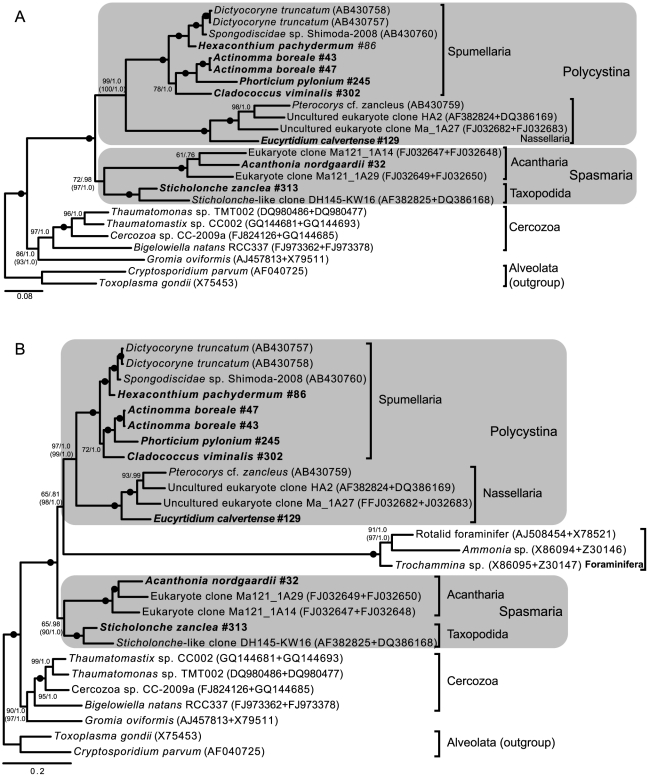
Maximum likelihood (ML) phylogeny of Radiolaria inferred from an 18S + 28S rDNA alignment consisting of A) 24 taxa and 4163 characters, and B) 27 taxa and 4163 characters (including Foraminifera). Values at nodes represent bootstrap support values from ML analysis and Bayesian posterior probabilities (pp) (ML/pp). Filled circle indicate maximum support in both analyses. Numbers in parenthesis represent the peak support values after removing fast evolving sites for selected nodes (cf. Table S3 and S4).

From 8 individuals (5 Spumellaria, 1 Nassellaria, 1 Acantharia and 1 Taxopodida) we sequenced both the 18S rDNA and the 28S rDNA gene (Table 1). The phylogeny generated from a concatenated dataset consisting of 27 taxa showed a different topology than the larger 18S rDNA phylogeny (Figure 3a). In contrast to the 18S rDNA tree, Polycystina and the two subgroups Nassellaria and Spumellaria formed highly supported clades (all groups >99%/1.0 pp); and Acantharia (100%/1.0 pp) and Taxopodida (100%/1.0 pp) clustered together as Spasmaria with moderate support (72%/0.98 pp). When removing fast evolving sites the support values for Spasmaria increased to 97%/1.0 (Table S3). When Foraminifera were included in the analyses they grouped with Radiolaria with maximum support (100%/1.0 pp; Figure 3b). Foraminifera clustered as sister to Polycystina with rather low support (65%/0.81 pp). However, the support for this sisterhood increased to 98%/1.0 when the 90% fastest sites were removed from the analysis (Table S4). *Gromia oviformis* was highly supported as sister to Cercozoa, also when Foraminifera was included.

Separate phylogenies were generated from each of the 18S and 28S genes in the 27 taxon alignment (Figures S1 and S2). The resulting 18S tree was very similar to the 18S phylogeny inferred from the 158 taxon alignment in figure 2, but Acantharia clustered as sister to Taxopodida and Spumellaria with weak support (63%). This position of Acantharia was is identical to the 18S tree from 158 taxa when fast evolving sites had been removed (Table S1). The single gene 28S tree showed essentially identical topology as in the combined 18S+28S tree and retrieved Polycystina and Spasmaria with high support (Fig. S2).

## Discussion

### 18S+28S rDNA phylogeny supports monophyletic Polycystina and Spasmaria

The Radiolaria 18S rDNA phylogeny presented here is similar to other recently published 18S rDNA trees [6], [7], [11], [20]. Spumellaria is not monophyletic as the colonial and naked spumellarians group robustly within Nassellaria, a clustering pattern consistently recovered with high support in 18S rDNA trees. Furthermore, Acantharia is weakly branching either as sister to Nassellaria or to Taxopodida and Spumellaria. The latter two are always clustering together suggesting Polycystina as a polyphyletic group. Phylogenies generated from the two 18S alignments were essentially identical, except Acantharia had an unstable position. However, when fast evolving sites were removed from the most taxon rich dataset, the two phylogenies were similar. This implies that the phylogeny inferred from the 18S gene is not substantially affected by differences in taxon sampling.

The concatenated analysis of 18S + 28S rDNA radically changed the Radiolaria phylogeny and received higher statistical support for most of the subgroups and deeply diverging branching points. In contrast to the 18S rDNA tree, the combined gene tree is in better accordance with morphological characteristics [1], [3] by retrieving monophyletic Polycystina (Nassellaria together with Spumellaria) and Acantharia as sister to Taxopodida – a clade earlier named Spasmaria [8]. Although relatively few Radiolaria species have been sequenced for the 28S gene, the data suggest that combining the 18S and 28S rDNA genes may improve the resolution of the Radiolaria phylogeny [see 34]. This revised phylogeny of Radiolaria has several impacts on the interpretation of cellular evolution of the group. Most importantly the concatenated phylogeny significantly simplifies the evolution of the central capsule and the skeleton structure.

### Central capsule evolution

The central capsule has been one of the most important characters uniting the Polycystina, Acantharia and Phaeodaria in the group of Radiolaria [2], [3]. But the importance of this feature has been challenged as molecular analyses have shown that phaeodarians, which do have a central capsule, belong to Cercozoa, while Taxopodida, without a central capsule, is strongly supported as one of the branches of Radiolaria [5], [35]. Although Taxopodida (*S. zanclea*) lacks a typical central capsule, it does possess a thick nuclear capsule with a rigid layer beneath the nuclear envelope into which the axopodia are inserted [36]. Cachon [37] refers to this as the central capsule, but concludes that it is not likely homologous to the central capsule found among radiolarians. The central capsule of Acantharia is described by Levine [38] as an inner envelope closely lining the central cell mass. If the main topology in the 18S+28S rDNA tree is confirmed by future studies, it implies that the typical radiolarian central capsule found in Spumellaria and Nassellaria was most likely invented only once before they divided into separate lineages, and that the central capsule of Acantharia and the nuclear capsule of Taxopodida might be related structures, possibly derived from the common ancestor of both Spasmaria and Polycystina.

### Skeletal evolution

In addition to the central capsule the mineral skeleton morphology has been an important character unifying the radiolarians and for traditional classification of radiolarian species [2], [3]. Polycystine radiolarians are either naked or have silica in their skeleton as opposed to acantharians which have strontium sulfate as the mineral in their skeleton [1], [39]. The polyphyly of Polycystina, as suggested by recent 18S rDNA phylogenies therefore indicated that the ability to produce silicate skeletons has arisen several times in Radiolaria. This was further supported by the inclusion of Taxopodida, which do not possess a skeleton as the other radiolarians, but do have bundles of siliceous spicules [36]. However, the monophyly of Polycystina shown by the 18S+28S rDNA phylogeny again brings together the siliceous skeleton bearing Spumellaria and Nassellaria, implying that this kind of skeleton likely originated only once in the common ancestor of both groups.

It has been observed that the swarmer cells produced by spumellarian species contain a single crystal of strontium sulfate [40], showing that this mineral is utilized by both this group and Acantharia. As the 18S+28S rDNA phylogeny divides these two groups, it is possible that the ability to accumulate strontium sulfate is an ancient feature of all Radiolaria that is now being used at different developmental stages in the cell cycle. As strontium crystals are observed in both naked and skeleton bearing solitary radiolarians, the ability to form a strontium crystal is however not necessarily related to the formation of strontium skeletons in acantharians [41].

### Acantharia and Taxopodida together as Spasmaria

The sister relationship between Acantharia and Taxopodida in the concatenated analysis is intriguing because the morphology of Taxopodida is very different from the other radiolarians and the group was for a long time classified among Heliozoa [38]. However there are some morphological features shared by Acantharia and Taxopodida which support their common ancestry. Both Acantharia and Taxopodida species possess myonemes, contractile organelles made of bundles of microfilaments that are used to generate movement of the axiopods in Taxopodida and the spicules of Acantharia [36], [42]. In Acantharia these are used for buoyancy control by regulating the cell volume [43]. In the taxopodidan *S. zanclea* the moving axopods function like oars with which the cell is able to move through the water masses [36]. Based on these similarities Cavalier-Smith [8] proposed the sub-phylum Spasmaria to include Acantharia and Taxopodida and this group seems now also to be supported by molecular data. Including Foraminifera in the 18S+28S rDNA phylogeny slightly reduced the support values for both Polycystina and Spasmaria. But these were again strongly supported when fast evolving sites were removed.

### 18S +28 rDNA phylogeny supports the Retaria hypothesis

When we added Foraminifera to our 18S and 18S+28S rDNA data both phylogenies grouped them with the radiolarians with high support (Figures 2 and 3b). This is similar to what has been observed in other phylogenies of 18S rDNA [6], [12], [17]. The name Retaria has been given to the group including Radiolaria and Foraminifera [17], [19]. But as the foraminifer sequences are known to generate very long branches in phylogenetic trees, especially due to their very aberrant 18S rDNA genes, this sister relationship has generally been regarded as a long branch artifact [12]. However, in a recent multigene phylogenomic analysis of Rhizaria Foraminifera clustered together with Acantharia with high support, providing strong support for the Retaria hypothesis [18]. But whether Foraminifera should be regarded as sister to Radiolaria or grouped within is still unresolved, mainly because the multigene data lack members from Polycystina.

Foraminifera and Radiolaria are both members of the supergroup Rhizaria, a group supported entirely by molecular data [12], [14]. The close relationship between Cercozoa and Foraminifera was established by molecular phylogeny using actin genes [44], and later also by 18S rDNA [45]. Independent evidence has been provided from analysis of “rare genomic changes”, i.e. an insertion in the polyubiquitin gene found in Cercozoa and Foraminifera, but not in Radiolaria [46], [47]. However, a recent EST survey identified such an insertion in Acantharia thereby questioning the significance of this marker [18].

Although our analysis of 18S and 28S rDNA cannot unambiguously solve the placement of Foraminifera, it suggests that they are sisters to Polycystina (Figure 3b) rather than to the entire radiolarian group, or to Spasmaria as suggested in Marande et al. [34]. When we removed the fastest evolving sites from the data the affiliation to Polycystina became substantially stronger, and gives better support to a position of Foraminifera within Radiolaria (Table S4). However, due to the rapid evolution of foraminiferan ribosomal genes this relationship should be confirmed by protein coding genes. In fact, a sister relationship can be regarded as a more parsimonious scenario considering that Foraminifera lack a central capsule and that their tests are very different from the radiolarian skeletons.

### Single cell whole genome amplification (SCWGA)

In this study we have successfully generated 18S rDNA sequences from 25 individuals of Nassellaria, Spumellaria, Taxopodida, Acantharia and Phaeodaria (Figure 1). In addition we have obtained a second genomic marker, 28S rDNA, from 8 individuals from Taxopodida, Acantharia, Nassellaria and solitary Spumellaria (Figure 3). To our knowledge no study aiming at obtaining molecular markers from single radiolarian and phaeodarian cells have had a similar success rate. The approach of combining SCWGA and gene-targeted PCR has recently been used with success to amplify and detect gene sequences from intracellular symbionts of radiolarians (unpublished results). Here we show that this method is very useful for obtaining suitable genetic material for phylogenetic reconstruction of Radiolaria.

## Supporting Information

Figure S1 **Maximum likelihood (ML) phylogeny of the 18S rDNA gene based on the alignment from the 18S+28S rDNA analysis (24 taxa and 1548 characters).** Values on nodes correspond to bootstrap support values. ML analyses performed as described in the manuscript.
(EPS)

Figure S2 **Maximum likelihood (ML) phylogeny of the 28S rDNA gene based on the alignment from the 18S+28S rDNA analysis (24 taxa and 2615 characters).** Values on nodes correspond to bootstrap support values. ML analyses performed as described in the manuscript. Grey boxes highlight the Polycystina and Spasmaria groups.
(EPS)

Table S1 **The Maximum Likelihood bootstrap support for important nodes in the 18S rDNA tree after removal of fast evolving sites.**
(DOC)

Table S2 **Support values for important nodes in the 18S rDNA tree after removing naked and colonial spumellarians (Collodaria).**
(DOC)

Table S3 **The support values (Maximum Likelihood bootstrap/Bayesian posterior probability) for important nodes in the 18S +28S rDNA phylogeny after removal of fast evolving sites with Foraminifera excluded from the analysis.**
(DOC)

Table S4 **The support values (Maximum Likelihood bootstrap/Bayesian posterior probability) for important nodes in the 18S +28S rDNA phylogeny after removal of fast evolving sites.**
(DOC)
