# Supplementary material for: Radiolaria Divided into Polycystina and Spasmaria in Combined 18S and 28S rDNA Phylogeny
Source: PLoS One. 2011 Aug 10;6(8):e23526. doi: 10.1371/journal.pone.0023526 (PMC3154480; doi:10.1371/journal.pone.0023526)
Supplement: Table S1 — The Maximum Likelihood bootstrap support for important nodes in the 18S rDNA tree after removal of fast evolving sites. (DOC) [file pone.0023526.s003.doc]

**Table S1**.

| **Sites removed (percentage of total distribution)** | **0** | **24 (10%)** | **27 (20%)** | **30 (30%)** | **35 (40%)** | **43 (50%)** | **92 (60%)** | **176 (70%)** | **282 (80%)** | **530 (90%)** |
| --- | --- | --- | --- | --- | --- | --- | --- | --- | --- | --- |
| Retaria | 98 | 97 | 98 | 98 | 98 | 98 | 97 | 95 | 92 | 73 |
| Cercozoa | 91 | 93 | 91 | 91 | 93 | 88 | 89 | 84 | 96 | 37 |
| Spumellaria | 93 | 91 | 90 | 91 | 89 | 86 | 91 | 98 | 89 | 40 |
| Taxopodida | 77 | 68 | 81 | 79 | 79 | 79 | 55 | 20 | 33 | 32 |
| Spumellaria + Taxopodida | 70 | 70 | 66 | 67 | 65 | 63 | 58 | 65 | 77 | 19 |
| Acantharia | 98 | 96 | 97 | 98 | 97 | 99 | 99 | 99 | 99 | 95 |
| Nassellaria (including Collodaria) | 100 | 100 | 100 | 100 | 100 | 100 | 100 | 100 | 100 | 100 |
| Acantharia +Nassellaria + Foraminifera | 42 | 39 | 37 | 38 | 36 | 36 | 33 | 50 | 0 | 27 |
| Acantharia +Spumellaria | 0 | 0 | 0 | 0 | 0 | 0 | 0 | 0 | 42 | 0 |
| Foraminifera | 100 | 100 | 100 | 100 | 100 | 100 | 100 | 100 | 100 | 100 |
| Foraminifera + Nassellaria | 56 | 54 | 51 | 54 | 50 | 53 | 53 | 67 | 76 | 49 |
| Collodaria | 99 | 100 | 100 | 100 | 100 | 100 | 100 | 100 | 100 | 99 |
