# Supplementary material for: Radiolaria Divided into Polycystina and Spasmaria in Combined 18S and 28S rDNA Phylogeny
Source: PLoS One. 2011 Aug 10;6(8):e23526. doi: 10.1371/journal.pone.0023526 (PMC3154480; doi:10.1371/journal.pone.0023526)
Supplement: Table S2 — Support values for important nodes in the 18S rDNA tree after removing naked and colonial spumellarians (Collodaria). (DOC) [file pone.0023526.s004.doc]

**Table S2**.

|  | ML value (%) | Bayes (pp) |
| --- | --- | --- |
| Retaria | 96 | 1.0 |
| Cercozoa | 93 | 1.0 |
| Spumellaria | 94 | 1.0 |
| Taxopodida | 82 | 1.0 |
| Spumellaria + Taxopodida | 77 | 1.0 |
| Acantharia | 98 | 1.0 |
| Nassellaria | 100 | 1.0 |
| Acantharia + Nassellaria + Foraminifera | 48 | 0.80 |
| Foraminifera | 100 | 1.0 |
| Foraminifera + Nassellaria | 66 | 0.97 |
