# Supplementary material for: Radiolaria Divided into Polycystina and Spasmaria in Combined 18S and 28S rDNA Phylogeny
Source: PLoS One. 2011 Aug 10;6(8):e23526. doi: 10.1371/journal.pone.0023526 (PMC3154480; doi:10.1371/journal.pone.0023526)
Supplement: Table S3 — The support values (Maximum Likelihood bootstrap/Bayesian posterior probability) for important nodes in the 18S +28S rDNA phylogeny after removal of fast evolving sites with Foraminifera excluded from the analysis. (DOC) [file pone.0023526.s005.doc]

**Table S3**.

| **Sites removed (percentage of total distribution)** | **0** | **164 (10%)** | **231 (20%)** | **293 (30%)** | **366 (40%)** | **460 (50%)** | **581 (60%)** | **781 (70%)** | **1092 (80%)** | **1641 (90%)** |
| --- | --- | --- | --- | --- | --- | --- | --- | --- | --- | --- |
| Polycystina | 99 | 99/1.0 | 99/1.0 | 99/1.0 | 100/1.0 | 100/1.0 | 100/1.0 | 100/1.0 | 100/1.0 | 100/1.0 |
| Spasmaria | 72 | 74/0.99 | 78/0.99 | 72/0.99 | 70/0.99 | 76/0.99 | 84/1.0 | 89/1.0 | 97/1.0 | 68/0.71 |
| Cercozoa | 86 | 90/1.0 | 93/1.0 | 89/1.0 | 86/1.0 | 89/1.0 | 83/1.0 | 74/0.98 | 93/1.0 | 76/1.0 |
