# Supplementary material for: Radiolaria Divided into Polycystina and Spasmaria in Combined 18S and 28S rDNA Phylogeny
Source: PLoS One. 2011 Aug 10;6(8):e23526. doi: 10.1371/journal.pone.0023526 (PMC3154480; doi:10.1371/journal.pone.0023526)
Supplement: Table S4 — The support values (Maximum Likelihood bootstrap/Bayesian posterior probability) for important nodes in the 18S +28S rDNA phylogeny after removal of fast evolving sites. (DOC) [file pone.0023526.s006.doc]

**Table S4**.

| **Sites removed (percentage of total distribution)** | **0** | **166 (10%)** | **219 (20%)** | **276 (30%)** | **324 (40%)** | **401 (50%)** | **528 (60%)** | **745 (70%)** | **1077 (80%)** | **1705 (90%)** |
| --- | --- | --- | --- | --- | --- | --- | --- | --- | --- | --- |
| Polycystina | 97 | 96/1.0 | 94/1.0 | 95/1.0 | 92/1.0 | 96/1.0 | 96/1.0 | 99/1.0 | 99/1.0 | 74/0.99 |
| Retaria | 100 | 100/1.0 | 100/1.0 | 100/1.0 | 100/1.0 | 100/1.0 | 100/1.0 | 100/1.0 | 100/1.0 | 100/1.0 |
| Spasmaria | 65 | 73/0.99 | 75/0.99 | 75/1.0 | 74/1.0 | 76/1.0 | 59/0.99 | 69/0.96 | 90/1.0 | 83/1.0 |
| Cercozoa | 90 | 91/1.0 | 87/1.0 | 89/1.0 | 88/1.0 | 84/1.0 | 85/1.0 | 77/1.0 | 97/1.0 | 85/1.0 |
| Foraminifera + Polycystina | 65 | 70/0.87 | 73/0.91 | 73/0.94 | 78/0.95 | 78/0.96 | 78/0.97 | 58/0.76 | 54/0.77 | 98/1.0 |
